# Maternal and cord blood vitamin D level and the infant gut microbiota in a birth cohort study

**DOI:** 10.1186/s40748-020-00119-x

**Published:** 2020-10-20

**Authors:** Zeinab Kassem, Alexandra Sitarik, Albert M. Levin, Susan V. Lynch, Suzanne Havstad, Kei Fujimura, Anita Kozyrskyj, Dennis R. Ownby, Christine Cole Johnson, Germaine J. M. Yong, Ganesa Wegienka, Andrea E. Cassidy-Bushrow

**Affiliations:** 1 Department of Public Health Sciences, Henry Ford Hospital, 1 Ford Place, 5C, Detroit, MI 48202 USA; 2 Department of Medicine, University of California, San Francisco, CA USA; 3 Department of Pediatrics, University of Alberta, Alberta, Canada; 4 Division of Allergy and Clinical Immunology, Department of Pediatrics, Georgia Regents University, Augusta, GA USA; 5 Center for Urban Responses to Environmental Stressors, Wayne State University, Detroit, MI USA

**Keywords:** Vitamin D, Gut microbiota, Birth cohort, Cord blood

## Abstract

**Background:**

Mounting evidence suggests both vitamin D and the early life gut microbiome influence childhood health outcomes. However, little is known about how these two important exposures are related. We aimed to examine associations between plasma 25-hydroxyvitamin D (25[OH]D) levels during pregnancy or at delivery (cord blood) and infant gut microbiota.

**Methods:**

Maternal and cord blood 25[OH]D levels were assessed in a sample of pregnant women. Compositional analyses adjusted for race were run on the gut microbiota of their offspring at 1 and 6 months of age.

**Results:**

Mean prenatal 25(OH)D level was 25.04 ± 11.62 ng/mL and mean cord blood 25(OH)D level was 10.88 ± 6.77 ng/mL. Increasing prenatal 25(OH)D level was significantly associated with decreased richness (*p* = 0.028) and diversity (*p* = 0.012) of the gut microbiota at 1 month of age. Both prenatal and cord 25(OH)D were significantly associated with 1 month microbiota composition. A total of 6 operational taxonomic units (OTUs) were significantly associated with prenatal 25(OH)D level (four positively and two negatively) while 11 OTUs were significantly associated with cord 25(OH)D (10 positively and one negatively). Of these, OTU 93 (*Acinetobacter*) and OTU 210 (*Corynebacterium*)*,* were consistently positively associated with maternal and cord 25(OH)D; OTU 64 (*Ruminococcus gnavus*) was positively associated with prenatal 25(OH)D but negatively associated with cord 25(OH)D.

**Conclusions:**

Prenatal maternal and cord blood 25(OH)D levels are associated with the early life gut microbiota. Future studies are needed to understand how vitamin D and the microbiome may interact to influence child health.

## Background

Vitamin D is necessary for optimal maternal and fetal health during pregnancy [1], however, vitamin D deficiency and inadequacy is common during this time [2]. In addition to potential bone problems in offspring, maternal vitamin D level may impact child health outcomes, including risk for allergy/asthma and obesity [3–5]. Growing evidence suggests these child health outcomes may also be associated with the gut microbiome [6].

Vitamin D level may impact the structure and function of the gut microbiome. In vitamin D receptor knockout mice, there are significant community level and functional changes in the gut microbiota compared to wild type mice [7, 8]. Mice that cannot produce 1,25-dihydroxycholecalciferol exhibit gut microbiome dysbioisis [8]. There is sufficient evidence in humans that vitamin D level is associated with gut microbiome composition [9]. Maternal dietary vitamin D intake is also associated with maternal gut microbiome measured 4 days after delivery [10]. Only a few studies have examined potential associations between maternal vitamin D level and gut microbiome in early life. In the KOALA birth cohort, maternal vitamin D supplementation and maternal 25-hydroxyvitamin D (25[OH]D) quintiles were negatively associated with counts of *Bifidobacterium* species and there was a positive association between maternal 25(OH)D quintiles and *B. fragilis* counts in 1 month old infant gut microbiota [11]. Cord blood 25(OH)D was associated with higher levels of *Lachnobacterium* and lower levels of *Lactococcus* in the Vitamin D Antenatal Asthma Reduction Trial [12]. These findings suggest maternal vitamin D levels might influence the abundance of several key bacterial taxa within the infant microbiota.

The infant gut microbiome is initially seeded by the maternal microbiome [13]; through an influence on the maternal microbiome, prenatal vitamin D level may potentially influence the infant gut microbiome. Additional study of the role of prenatal vitamin D in infant microbiome development is needed [12]. We examined if 25(OH)D levels during pregnancy (measured between 25 and 44 weeks gestation; mean = 33 weeks) or at delivery (in cord blood) were associated with the infant gut microbiota at infant ages ~ 1 month and ~ 6 months in a sample of maternal-child pairs from the racially and socioeconomically diverse Wayne County Health, Environment, Allergy and Asthma Longitudinal Study (WHEALS) birth cohort [3, 14].

## Methods

### Study population

WHEALS recruited pregnant women with due dates from September 2003 through December 2007, and who were seeing an obstetric practitioner at 1 of 5 clinics of an urban health system to establish a birth cohort [3, 14]. All women were in their second trimester or later, were aged 21–49 years, and were living in a predefined geographic area in Wayne and Oakland counties that included the city of Detroit as well as the suburban areas immediately surrounding the city. All participants provided written, informed consent and study protocols were approved by the Institutional Review Board at Henry Ford Health System. The original WHEALS cohort recruited 1258 mother-child pairs.

### Stool specimens

Home visits with participants were conducted targeting infant ages 1 and 6 months. Families were asked to retain the most recent soiled diaper prior to the home visit and stool samples were banked at − 80 °C. Detailed information on DNA extraction methods are presented elsewhere [15].

### Polymerase chain reaction conditions and library preparation for sequencing

The V4 region of the 16S rRNA gene was amplified, as described by Caporaso, Lauber [16]. Briefly, 16S rRNA amplification was performed in 25-μL reactions using 0.025 U Takara Hot Start ExTaq (Takara Mirus Bio Inc., Madison, WI), 1X Takara buffer with MgCl2, 0.4 pmol/μL of F515 and R806 primers, 0.56 mg/mL of bovine serum albumin (Roche Applied Science, Indianapolis, IN), 200 μM of dNTPs and 10 ng of genomic DNA. Reactions were performed in triplicate with the following: initial denaturation (98 °C, 2 min), 30 cycles of 98 °C (20 s), annealing at 50 °C (30 s), extension at 72 °C (45 s), and final extension at 72 °C (10 min). Amplicons were verified using a 2% Tris/Borate/EDTA agarose e-gel (Life Technologies, Grand Island, NY), cleaned and normalized using SequalPrep Normalization Plates (Applied Biosystems, Foster City, CA), and further quantified using the Qubit 2.0 Fluorometer and the double-stranded DNA HS Assay Kit (Life Technologies). Samples were pooled in equal moles at concentrations of 5 ng, purified using AMPure SPRI beads (Beckman Coulter, Brea, CA), denatured and diluted to 2 nM, and 5 pM was loaded onto the Illumina Nextseq cartridge with 40% (v/v) of denatured 12.5 pM PhiX spike-in control.

### Sequence data processing and quality control

Paired-end sequences were assembled using FLASH v 1.2.7 [17], de-multiplexed by barcode, and low quality reads (Q-score < 30) were discarded in QIIME 1.8 [18]. Reads were truncated if 3 consecutive bases were < Q30, and were retained only if the truncated sequence was ≥75% of the original length. UCHIME [19] was used to check for chimeras, which were filtered from the dataset prior to operational taxonomic unit (OTU) picking at 97% sequence identification using UCLUST [20] against the GreenGenes database version 13_5 [21]; sequence reads that failed to cluster with a reference sequence were clustered de novo. Sequences were aligned using PyNAST [22], and taxonomy assigned using the RDP classifier [23] and GreenGenes reference database version 13_5 [21]. FastTree 2.1.3 [24] was used to build a phylogenetic tree. To normalize variation in read depth across samples, data was rarefied to the minimum read depth of 60,000 sequences per sample. To ensure that a representative subsample was selected, a representative rarefying algorithm described previously was implemented [25].

A total of 580 children had at least 1 stool sample in the final rarefied OTU table; of these, 499 unique children (700 total stool specimens) had a maternal or cord blood 25(OH)D measurement and are included in the statistical analysis. Table 1 presents the breakdown of those with each measure (microbiota, 25[OH]D) by each time point (1 and 6 months or prenatal and cord, respectively). In the analytical dataset, stool specimens from the 1 month visit were collected at a mean ± standard deviation (SD) of 40 ± 17 days (minimum = 16, maximum = 137) and stool specimens from the 6 month visit were collected at a mean ± SD of 207 ± 31 days (minimum = 168, maximum = 322). Throughout, “1 month” and “6 month” are used as labels of the intended time period of sample collection.

**Table 1:** Number of mother-child pairs by vitamin D and fecal data time points (data as N [%])

	Fecal collection time points		
Vitamin D sample	1 month	6 months	Both
Prenatal	53 (31.2%)	44 (34.4%)	61 (30.4%)
Cord	31 (18.2%)	29 (22.6%)	36 (17.9%)
Both	86 (50.6%)	55 (43.0%)	104 (51.7%)

### Vitamin D measurement

25(OH)D was measured in frozen (− 80 °C) plasma samples from pregnancy (range = 25–44 weeks gestation; mean = 33 weeks) and delivery (cord blood) in the laboratory of Dr. Neil Binkley at the University of Wisconsin using a high-performance liquid chromatography method [3, 14, 26]. For those with 25(OH)D levels below the lowest detectable limit of 5 ng/mL, a value of 2.5 ng/mL was assigned (*N* = 11 for prenatal and *N* = 81 for cord 25[OH]D). There is no overall consensus on optimal vitamin D levels in pregnancy; however, levels above 20 ng/mL prevent bone-related pathologies [2]. Therefore, insufficient levels of vitamin D were defined as < 20 ng/mL [2].

To account for seasonal variation in vitamin D, as described by Wegienka, Havstad [3], we fit a sinusoidal model of the values (25[OH]D value) and time (month, denoted as “m”) of collection:

$$ 25\left(\mathrm{OH}\right)\mathrm{D}\ \mathrm{level}={\beta}_0+{\beta}_1\sin\ \left(2\pi \mathrm{m}/12\right)+{\beta}_2\cos\ \left(2\pi \mathrm{m}/12\right). $$

Deseasonalized values [27] were calculated by taking each subject’s measured value, subtracting the predicted value and adding back the overall mean. Deseasonalized values were used for analysis.

### Statistical analysis

Significance for main effects was pre-specified at *p* < 0.05 and for interaction effects at *p* < 0.1. Compositional differences in the gut microbiota by maternal and cord blood 25(OH)D measures were assessed by permutational multivariate analysis of variance as implemented in the R package *“vegan”* [28], using both weighted and unweighted UniFrac [29]. Alpha diversity indices (richness, Pielou’s evenness, and Faith’s phylogenetic diversity) were calculated using QIIME [18] and the R vegan package [28], and tested for associations with maternal and cord blood 25(OH)D using linear regression. Individual taxa tests were conducted on all OTUs found in 10% or more of samples using zero-inflated negative binomial regression (or the standard negative binomial if convergence failed). *P*-values were corrected using the Benjamini and Hochberg [30] false discovery rate; false discovery rate adjusted *p* < 0.05 was considered significant. We a priori hypothesized that race may confound and/or modify associations between maternal and cord blood 25(OH)D and infant gut microbiota, thus all analyses were adjusted for maternal race (Black versus White, excluding others) and we also tested for race-specific effects with stratified models and interaction terms.

Finally, as breastfeeding is associated with early-life gut microbiota [15] and with lower levels of vitamin D in infancy [31], we hypothesized that breastfeeding could modify associations of maternal and cord blood 25(OH)D with gut microbiota. For each microbiota time-point (1 or 6 months), current breastfeeding was defined as any current breastfeeding at that timepoint. Interaction terms were fit between maternal or cord 25(OH)D and current breastfeeding to examine potential effect modification.

## Results

### Basic descriptives

Table 2 presents demographic information comparing characteristics of those included in the analytic sample to those not included. Compared to those not included in the sample, those included were slightly older, and were more often White, married, non-urban dwelling, had higher incomes and were less likely to be exposed to environmental tobacco smoke prenatally (all *p* < 0.05). Mean birth weight Z-score of children in the analytic sample was also higher.

**Table 2:** Characteristics of participants included and excluded from analysis (data as mean ± SD or N (%))

	Included in analysis subset		
Covariate	No (*N* = 759)	Yes (*N* = 499)	*p*-value^*^
Maternal age (years)	29.2 **±** 5.2	30.1 **±** 5.2	**0.002**
Maternal race			
White	150 (19.8%)	140 (28.1%)	**0.001**
Black	497 (65.5%)	281 (56.3%)	
Other	112 (14.8%)	78 (15.6%)	
Mother married			
No	318 (41.9%)	167 (33.5%)	**0.003**
Yes	441 (58.1%)	332 (66.5%)	
Urban residence			
No	299 (39.4%)	256 (51.3%)	**<.001**
Yes	460 (60.6%)	243 (48.7%)	
Household income			
< $40,000	328 (43.2%)	149 (29.9%)	**<.001**
$40,000–$80,000	210 (27.7%)	137 (27.5%)	
$80,000+	139 (18.3%)	144 (28.9%)	
Refused/did not answer	82 (10.8%)	69 (13.8%)	
Gestational age^a^	38.7 ± 1.8	38.8 ± 1.6	0.27
Birthweight z-score^b^	−0.196 ± 0.972	− 0.005 ± 1.004	**0.001**
First born child			
No	492 (64.8%)	306 (61.3%)	0.21
Yes	267 (35.2%)	193 (38.7%)	
Mode of delivery			
Vaginal	456 (60.6%)	328 (65.9%)	0.061
C-Section	296 (39.4%)	170 (34.1%)	
Season of birth			
Winter	156 (20.6%)	101 (20.2%)	0.53
Spring	182 (23.9%)	103 (20.6%)	
Summer	202 (26.6%)	143 (28.7%)	
Fall	219 (28.9%)	152 (30.5%)	
Environmental tobacco smoke at pre-delivery			
No	517 (68.1%)	394 (79%)	**<.001**
Yes	242 (31.9%)	105 (21%)	

^*^Calculated by analysis of variance for numerical covariates and chi-square test for categorical covariates

^a^*N* = 737 for No and *N* = 495 for Yes

^b^*N* = 700 for No and *N* = 472 for Yes

Mean prenatal 25(OH)D was 25.04 ± 11.62 ng/mL, while mean cord blood 25(OH)D was 10.88 ± 6.77 ng/mL. Among the 403 mothers with prenatal 25(OH)D measurements, 141 (35%) had insufficient prenatal vitamin D (25[OH]D < 20 ng/mL). Prenatal and cord 25(OH)D were highly correlated (Pearson ρ = 0.81, *p* < 0.001).

### Association of prenatal and cord blood 25(OH)D with gut microbiota alpha diversity

After adjusting for race, higher prenatal 25(OH)D level was significantly associated with decreased richness (*p* = 0.028) and diversity (*p* = 0.012) of the gut microbiota at 1 month of age. There were no other main effects of prenatal or cord 25(OH)D levels on the infant gut microbiota at 1 or 6 months of age after adjusting for race (Table 3).

**Table 3:** Alpha diversity metrics by deseasonalized maternal Vitamin D, overall and by race

Outcome	Model	β^a^	SE	*p*-value
**1-Month**				
**Prenatal Vitamin D**				
Richness	Overall (unadjusted)	−4.872	1.201	**<.001**
	Overall (adjusted for race)^b^	−3.293	1.488	**0.028**
	Black	−4.409	1.912	**0.022**
	White	−1.548	2.371	0.515
	Interaction *p*-value			0.35
Evenness	Overall (unadjusted)	−0.004	0.002	**0.046**
	Overall (adjusted for race)^b^	− 0.002	0.003	0.498
	Black	−0.004	0.003	0.26
	White	0.001	0.004	0.76
	Interaction *p*-value			0.347
Diversity	Overall (unadjusted)	−0.293	0.063	**<.001**
	Overall (adjusted for race)^b^	−0.199	0.079	**0.012**
	Black	−0.281	0.101	**0.006**
	White	−0.071	0.125	0.567
	Interaction *p*-value			0.195
**Cord Vitamin D**				
Richness	Overall (unadjusted)	−6.363	2.306	**0.006**
	Overall (adjusted for race)^b^	−2.001	2.660	0.453
	Black	−5.955	3.455	0.087
	White	3.516	4.126	0.397
	Interaction *p*-value			**0.079**
Evenness	Overall (unadjusted)	−0.005	0.004	0.219
	Overall (adjusted for race)^b^	0.002	0.005	0.712
	Black	−0.009	0.007	0.194
	White	0.017	0.008	**0.044**
	Interaction *p*-value			**0.016**
Diversity	Overall (unadjusted)	−0.385	0.122	**0.002**
	Overall (adjusted for race)^b^	−0.174	0.142	0.223
	Black	−0.370	0.191	0.055
	White	0.100	0.206	0.629
	Interaction *p*-value			0.103
**6-Months**				
**Prenatal Vitamin D**				
Richness	Overall (unadjusted)	−4.447	1.513	**0.004**
	Overall (adjusted for race)^b^	−2.324	1.852	0.211
	Black	−2.364	2.590	0.363
	White	−2.276	2.626	0.388
	Interaction *p*-value			0.981
Evenness	Overall (unadjusted)	−0.004	0.002	0.097
	Overall (adjusted for race)^b^	−0.001	0.003	0.643
	Black	0.000	0.004	0.973
	White	−0.003	0.005	0.553
	Interaction *p*-value			0.648
Diversity	Overall (unadjusted)	−0.229	0.075	**0.003**
	Overall (adjusted for race)^b^	−0.116	0.091	0.206
	Black	−0.167	0.128	0.195
	White	−0.055	0.128	0.667
	Interaction *p*-value			0.544
**Cord Vitamin D**				
Richness	Overall (unadjusted)	−8.437	2.860	**0.004**
	Overall (adjusted for race)^b^	−6.181	3.300	0.063
	Black	−10.201	4.241	**0.018**
	White	0.673	5.153	0.897
	Interaction *p*-value			0.112
Evenness	Overall (unadjusted)	−0.007	0.005	0.166
	Overall (adjusted for race)^b^	−0.008	0.006	0.177
	Black	−0.017	0.007	**0.025**
	White	0.007	0.010	0.475
	Interaction *p*-value			**0.053**
Diversity	Overall (unadjusted)	−0.409	0.140	**0.004**
	Overall (adjusted for race)^b^	−0.300	0.159	0.061
	Black	−0.479	0.205	**0.021**
	White	0.006	0.248	0.979
	Interaction p-value			0.141

*SE* standard error

^a^Estimated change in alpha diversity measure for a 5 ng/mL increase in vitamin D

^b^Restricted to Black or White only, so reduced sample size compared to unadjusted

There was evidence that race modified associations between cord blood 25(OH)D and alpha diversity metrics of the infant gut microbiota at 1 and 6 months (Table 3). There was evidence for a race-specific effect between cord 25(OH)D level and microbial evenness at 1-month (interaction *p* = 0.016), where higher cord 25(OH)D level was associated with higher infant gut evenness, but only among White women (*p* = 0.044). Although there was evidence of a race-specific effect of cord 25(OH)D on richness at 1 month (interaction *p* = 0.079) with higher cord 25(OH)D inversely associated with richness only in Blacks; in models stratified by race this effect was not statistically significant. Race also modified the association of cord 25(OH)D with infant gut evenness at the 6 month visit (interaction *p* = 0.053); in Black women, higher cord 25(OH)D was associated with decreased infant gut evenness (*p* = 0.025) but there was no association in White children (*p* = 0.475).

There was no evidence current breastfeeding modified associations of maternal or cord 25(OH)D and 1- or 6-month infant gut microbiota alpha diversity metrics (all interaction *p* > 0.42).

### Association of prenatal and cord blood vitamin D with gut microbiota composition

After adjusting for race, both prenatal (*p* = 0.029 for unweighted UniFrac, *p* = 0.030 for weighted UniFrac) and cord 25(OH)D (*p* = 0.028 for unweighted UniFrac, *p* = 0.044 for weighted UniFrac) levels were significantly associated with 1-month microbiota composition, though only a small proportion of variability in microbiota composition was explained by maternal or cord vitamin D (Table 4). No significant associations were found between prenatal and cord 25(OH)D levels and microbiota composition at 6 months of age, after adjusting for race (Table 4). There was evidence of a race-specific effect of prenatal 25(OH)D on 1-month infant gut microbiota composition (interaction *p* = 0.089), with associations in Black (*p* = 0.006 for weighted UniFrac) but not White women (*p* = 0.375 for weighted UniFrac). There was no evidence that current breastfeeding modified associations of maternal or cord 25(OH)D and 1- or 6-month infant gut microbiota composition (all interaction *p* > 0.40).

**Table 4:** Compositional differences by deseasonalized maternal vitamin D, overall and by race

	1-Month				6-Month			
	Unweighted UniFrac		Weighted UniFrac		Unweighted UniFrac		Weighted UniFrac	
	*p*-value	*R*^*2*^	*p*-value	*R*^*2*^	*p*-value	*R*^*2*^	*p*-value	*R*^*2*^
**Prenatal Vitamin D**								
Overall (unadjusted)	**< 0.001**	0.013	**0.042**	0.008	**0.001**	0.009	0.490	0.003
Overall (adjusted for race)^a^	**0.029**	0.006	**0.030**	0.011	0.212	0.005	0.719	0.002
Black	**0.001**	0.014	**0.006**	0.023	**0.037**	0.011	0.973	0.001
White	0.903	0.008	0.375	0.012	0.609	0.01	0.614	0.008
Interaction *p*-value	0.157		**0.089**		0.144		0.871	
**Cord Vitamin D**								
Overall (unadjusted)	**0.001**	0.009	0.097	0.007	**0.001**	0.01	0.690	0.003
Overall (adjusted for race)^a^	**0.028**	0.007	**0.044**	0.011	0.234	0.006	0.938	0.001
Black	**0.006**	0.014	0.053	0.016	**0.032**	0.013	0.697	0.005
White	0.782	0.011	0.442	0.013	0.956	0.010	0.805	0.007
Interaction *p*-value	0.292		0.516		0.350		0.527	

^a^Restricted to Black or White only, so reduced sample size compared to unadjusted

**Fig. 1:**
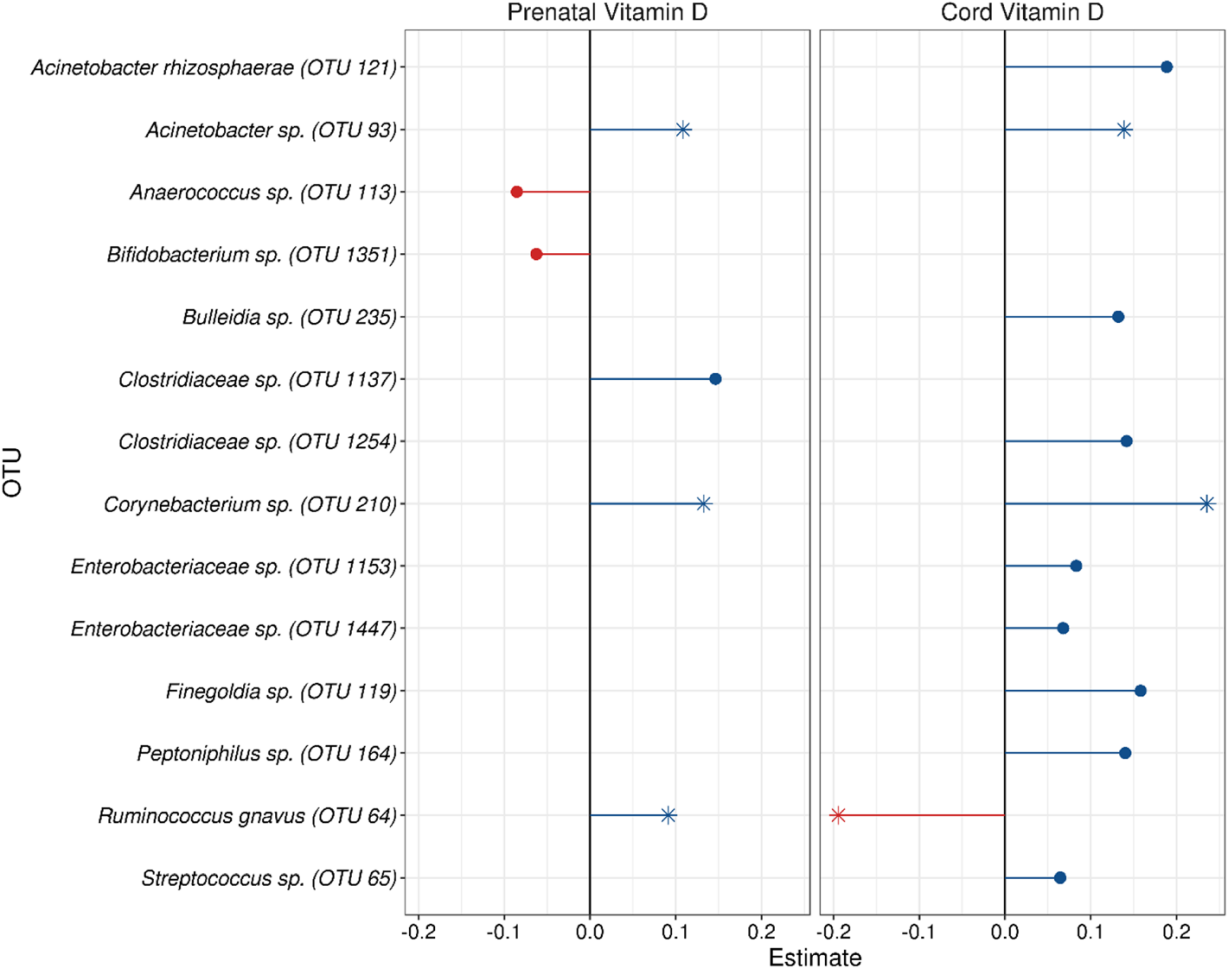
Association between deseasonalized vitamin D (prenatal and cord) and 1-month specific OTUs, after adjusting for maternal race. Plot displays all significant taxa (false discovery rate adjusted *p* < 0.05). OTUs significantly associated with both prenatal and cord vitamin D are indicated by an asterisk (*). The y-axis displays the specific OTU and the x-axis is the negative binomial regression coefficient. OTUs are colored by direction of association (blue (right side) = positive association, red (left side) = negative association). Abbreviations: OTU, operational taxonomic units

Based on compositional difference testing, individual OTU tests were conducted to associate deseasonalized prenatal and cord 25(OH)D level with 1-month infant gut OTUs only, after adjusting for maternal race (Fig. 1). In the 1-month samples, a total of 6 OTUs were significantly associated with prenatal 25(OH)D levels while 11 OTUs were significantly associated with cord 25(OH)D. The majority of significant OTUs were positively associated with prenatal and cord 25(OH)D levels (4/6 for prenatal vitamin D; 10/11 for cord vitamin D). Three OTUs were significantly associated with both prenatal and cord 25(OH)D: OTU 93 (*Acinetobacter*) and OTU 210 (*Corynebacterium*)*,* which were consistently positively associated with both prenatal and cord 25(OH)D, as well as OTU 64 (*Ruminococcus gnavus*), which was positively associated with prenatal 25(OH)D, but negatively associated with cord 25(OH)D.

## Discussion

In this racially diverse unselected birth cohort, we found evidence that prenatal and cord blood vitamin D levels were associated with early life (~ 1 month) gut microbiota. Conversely, there was no association of prenatal or cord blood vitamin D and gut microbiota measured at 6 months of age. This work extends that of previous studies of maternal vitamin D and the infant gut microbiome [11, 12] and provides new data on the associations of cord blood vitamin D level and the infant gut microbiome.

While some associations were unique, there were 3 OTUs in the 1 month gut microbiota significantly associated with both prenatal and cord blood 25(OH)D. Prenatal and cord blood 25(OH)D were both positively associated with *Acinetobacter* and *Corynebacterium* OTUs; in contrast, a *Ruminococcus gnavus* OTU was positively associated with prenatal 25(OH)D but negatively associated with cord blood 25(OH)D. Similar to our findings, Lundgren, Madan [32] found that *Acinetobacter* in stool samples (collected at 6 weeks) of newborns delivered via C-section was positively associated with maternal dairy intake, presuming increased maternal dairy intake is associated with higher vitamin D. Ooi, Li [8] found that defective vitamin D receptors in mice were associated with lower *Ruminococcaceae,* which is consistent with our findings with prenatal but not cord blood 25(OH)D.

Only 2 other studies have compared prenatal vitamin D level with infant gut microbiome. The KOALA Dutch cohort study composed of 913 mother-infant pairs assessed the association between prenatal 25(OH)D levels with the abundance of predefined bacterial taxa (*Bifidobacteria, Escherichia coli, Bacteroides fragilis, Clostridium difficile, and Lactobacilli*) in stool samples of children at 1 month of age [11]. Maternal 25(OH)D quintiles were negatively associated with counts of *Bifidobacterium* species but positively associated with counts of *B. fragilis* [11]. Similarly, prenatal 25(OH)D levels in WHEALS were inversely associated with *Bifidobacterium* species at 1 month. In contrast to our study, which utilized 16S rRNA V4 sequencing and did not a priori target specific bacterial taxa, in the KOALA cohort only specific bacterial groups were measured, thus relationships between 25(OH)D and other gut bacteria not measured in that study may have been missed. In a study population of 333 ethnically diverse mother-infant pairs that were part of a larger clinical trial, cord 25(OH)D levels were associated with increased levels of *Lachnospiraceae* and unclassified *Clostridiales* but decreased levels of *Lactococcus* [12]. In contrast to the unselected WHEALS cohort, this population was composed of children at higher risk for asthma.

Mechanistically, vitamin D could potentially impact gut microbiome structure and function via several mechanisms. Vitamin D receptor knockout status influences homeostasis in the intestines and gut microbiome of mice [7]. The active form of vitamin D (1,25-dihydroxycholecalciferol) and vitamin D receptor knockouts can affect the gut microbiome indirectly by reducing inflammation; gut inflammation provides pathogens with substrates that allow them to proliferate at the expense of more beneficial bacterial species [8]. Vitamin D may also impact the gut microbiome by upregulating innate immunity, producing antimicrobial peptides by macrophages, maintaining the function of the intestinal barrier and by altering calcium and phosphate absorption [33]. Additional research on potential mechanisms by which vitamin D influences the gut microbiome are needed.

In the current study, there were some inconsistent findings across the different time points of measurement. During pregnancy, the fetus’s only source of vitamin D is via the mother. However, after birth, the child transitions to obtaining vitamin D from direct sun exposure, dietary supplements and food (breastfeeding or formula feeding). Around the time of WHEALS births, in 2003, the American Academy of Pediatrics recommendations were that all breastfed infants, or non-breastfed infants who received less than 500 mL of vitamin D-fortified formula or milk, be given 400 IU of vitamin D per day [34]. We do not have information on whether or not the WHEALS children were supplemented with vitamin D in early life, thus we are unable to account for this potential postnatal factor. In WHEALS, although the association between prenatal and cord blood 25(OH)D is strong (*r* = 0.75), when maternal 25(OH)D is below a certain threshold (< 15 ng/mL), the correlation is weaker (*r* = 0.16) [14]. These findings suggest there exists a maternal vitamin D threshold below which mothers may insufficiently contribute to cord blood 25(OH)D and could partially explain why prenatal and cord 25(OH)D did not identically impact the infant gut microbiota.

We found evidence for race-specific effects of 25(OH)D on the infant microbiota. Studies have shown vitamin D levels vary among race groups, and this extends to the neonate [35]. Further, the correlation of prenatal and cord blood 25(OH)D is weaker among Black than White children (*r* = 0.65 and *r* = 0.87 respectively) [36]. Lower 25(OH)D levels among Blacks as compared to Whites can be explained by several factors. Darker skin pigmentation and thickness reduces levels of vitamin D production in the skin, a major source of vitamin D in the body [37, 38]. In general, Blacks consume less dietary vitamin D and milk products compared to Whites [37]. These racial differences can also be explained by genetic factors. Blacks have lower levels of vitamin D-binding protein, a serum transport protein, which contributes to lower levels of vitamin D [38]. Future studies are needed to better understand mechanisms, including genetic, dietary and/or cultural factors, that may explain why race modifies the association of 25(OH)D and the infant gut microbiota.

Given that we found associations between maternal and cord blood vitamin D and the infant gut microbiota at 1, but not 6, months of age, it is possible that maternal vitamin D level influences long-term offspring health through mechanisms other than the gut microbiome. Besides the microbiome, alternative mechanisms through which maternal vitamin D may influence child health includes suppression of inflammation [39] or epigenetic alternation [40].

In addition to future studies of these mechanisms, future studies should also consider obtaining meconium as a biospecimen for measuring the gut microbiome for additional studies on the impact of maternal vitamin D level and infant gut microbiome more proximal to the time of seeding.

Our study has a number of strengths and limitations. Our sample has considerable racial, educational and socioeconomic diversity and the early timeframe at which stool samples were collected (1 and 6 months), which allows for examination of the infant gut microbiome early on, presumably before external environmental exposure (i.e., diet diversity) has major impacts on composition. However, stool samples were only collected at 2 time points; given the rapidity of the development of the gut microbiome after birth, future studies should collect more samples over the first year of life. We do not have data on the infants’ vitamin D levels after birth nor do we have data on infants’ use of supplements after birth. However, because the vitamin D levels were measured prenatally and in cord blood, before the time of stool sample collection for gut microbiota measurement, it is unlikely that our results are due to reverse causality. There were significant differences between the participants that were included and excluded from the analytical sample, thus our results may be subject to selection bias.

## Conclusion

Prenatal maternal blood and cord blood 25(OH)D levels are associated with the very early life gut microbiota. Maternal vitamin D levels during pregnancy are associated with health in childhood, including obesity [4], allergy, and asthma [3]. Similar to vitamin D, the gut microbiome influences human health and disease and has also been shown to be associated with obesity [41], allergy, and asthma [25]. Future studies should examine if the gut microbiome mediates associations between vitamin D and disease or if the gut microbiome and vitamin D may interact to influence health and disease.

## Data Availability

The datasets used and/or analyzed during the current study are available from the corresponding author on reasonable request and with appropriate approvals.

## Abbreviations

25[OH]D: 25-hydroxyvitamin D
OTU: Operational taxonomic unit
SD: Standard deviation
WHEALS: Wayne County Health, Environment, Allergy and Asthma Longitudinal Study
